# Learning by exclusion in individuals with autism and Down syndrome

**DOI:** 10.1186/s41155-017-0064-x

**Published:** 2017-05-08

**Authors:** Luiza Costa Langsdorff, Camila Domeniconi, Andréia Schmidt, Camila Graciella Gomes, Deisy das Graças de Souza

**Affiliations:** Universidade Federal de Sao Carlos, Sao Carlos, Brazil

**Keywords:** Conditional discrimination, Auditory-visual relations, Exclusion trials, Autism, Down syndrome, Teaching technology

## Abstract

This study aimed to investigate the number of exclusion trials necessary for teaching auditory-visual relationships to individuals with autism and Down syndrome. Study participants were seven individuals with autism and a history of early behavioral intervention (EI), four adults with autism without a history of early behavioral intervention (NI), and three adults with Down syndrome. A set of procedures was used for teaching the auditory-visual matching to sample, and naming responses of the new stimuli were tested. For the individuals with autism and EI and for the individuals with Down syndrome, the required number of repetitions was stable and concentrated in the minimum programmed by the procedure (two repetitions). However, the procedure was not effective for teaching new conditional relationships for the adults with autism and NI. The results indicate that the procedure can constitute an important teaching technology; however, its efficacy appears to vary depending on the educational profile of the participant.

## Background

The term responding by exclusion (Dixon, 1977) has been used to describe the occurrence of conditional discrimination in which an undefined stimulus (i.e., not previously related to any other stimulus) is presented as the sample in matching to sample trials containing an also undefined comparison stimulus and at least one other previously defined comparison stimulus (i.e., previously associated with a different sample to that presented at the time). In this context, humans systematically choose the undefined comparison stimulus, *excluding* the one previously related with a different sample to the one presented in the current trial (e.g., Bates, 1979; Costa, McIlvane, Wilkinson, & de Souza, 2001; Dixon, 1977; Domeniconi, Costa, de Souza, & de Rose, 2007; Grassmann, Stracke, & Tomasello, 2009; Grassmann, Schulze, & Tomasello, 2015; McIlvane, Bass, O’Brien, Gerovac, & Stoddard, 1984; McIlvane, Klendaras, Lowry, & Stoddard, 1992; Stromer & Osborne, 1982; Wilkinson, Rosenquist, & McIlvane, 2009). Wilkinson, de Souza, and McIlvane (2000) indicate that this performance is widely replicable, with diverse populations of different development levels.

Despite the consistent data of Dixon’s study (1977) in exclusion probes, no evidence was found for stable learning of the relations in discrimination probes conducted after the exclusion trials. Further studies with different populations found that the learning of conditional relation between stimuli after a small number of exposures to exclusion trials is unlikely, even in individuals with typical development (Costa et al., 2001; Domeniconi et al., 2007; Wilkinson & McIlvane, 1997).

McIlvane and Stoddard (1981) investigated contingency arrangements necessary for the development of a teaching procedure that would guarantee the learning of conditional relations from exclusion trials. A young man with profound intellectual disabilities learned to select food items when the names of these foods were dictated in matching to sample tasks. In exclusion trials, new items were presented together with an already known item, and the participant systematically selected the unknown item when presented with the new name. After a history of reinforcement of these exclusion trials, the participant was able to demonstrate stable learning that was virtually error free for all the new items taught through this procedure. The authors attributed the very successful performance to the establishment of skills considered prerequisites for the task, such as differential control by auditory stimuli and a consistent baseline, capable of sustaining the performance by exclusion.

The role of differential consequences for accurate performance in exclusion trials was evaluated by Carr (2003) in an experiment that investigated whether the reinforcement contingent on correct responses in exclusion trials would strengthen the performance of relating new stimuli, in addition to improving the learning of these relations. Two experiments were conducted. The first experiment, conducted with seven children with autism, aged 3 to 6 years, tested the occurrence of responding by exclusion and the establishment of new auditory-visual relations between words and color photographs of objects, using reinforcers according to the child’s participation in the activity. In the second experiment, conducted with six children who did not respond by exclusion or did not demonstrate learning in the first experiment, training was scheduled in which correct responses in exclusion trials were differentially reinforced. Incorrect responses were followed by the introduction of a correction procedure. Only one child, among the seven participants, demonstrated consistent responding by exclusion and stable learning of relations between new stimuli in the first experiment. In the second experiment, five children consistently presented responding by exclusion and four presented improvements in learning of the new relations tested. The author argues that the use of reinforcers contingent on responses by exclusion can be favorable, both for the stabilization of this behavior as a generalized operant class—as the procedure encourages the selection of these items over that of familiar stimuli, as well as for learning of new relations between undefined stimuli (Carr, 2003).

Differential consequences for accurate performance in exclusion trials were also used by Ferrari, de Rose, and Mc Ilvane (1993, 2008), who obtained data similar to those of Carr (2003) regarding the efficiency of establishing relations between stimuli via reinforced exclusion trials. However, their experimental designs did not allow clarification of the number of exposures to this type of trial required for the consistent establishment of conditional relations between stimuli. In these studies, each new relation was presented by a variable number of exclusion trials before the application of the learning tests, with it being difficult to estimate how many trials were, in fact, necessary. Furthermore, the only learning test of the new relations occurred in selection probes (auditory-visual matching), with other performances that could possibly be learned from the exposure to the procedure not being investigated.

McIlvane et al. (1984) investigated whether a young man with intellectual disabilities would be able to name items learned in exclusion trials. According to the authors, positive performances in naming probes could more clearly evidence the learning of the relations taught, expanding the data obtained in learning probes that only require participants to selection responses in matching to sample trials, which is common in experiments using learning by exclusion (e.g., McIlvane & Stoddard, 1981; Dixon, 1977). Relations between food and their names were taught (some conventional names and other arbitrary ones). Three auditory-visual relations between foods and their names (already known to the participant) were used as defined samples and comparisons in the procedure, and 10 other relations were taught, in reinforced exclusion trials, with two comparisons available (one defined and the other undefined). Naming probes for the new foods were presented after every two exclusion trials.

The participant responded correctly to all the exclusion trials, maintaining accurate performance in the matching trials with the new stimuli, even when these relations were incorporated into the baseline. The naming of new foods occurred after a variable number of exclusion trials, ranging from two to 10. This study was important to show that, given adequate baseline conditions and the necessary prerequisites guaranteed, the emergency of naming can occur even without the direct teaching of this performance, after a teaching by exclusion procedure. Similar results were later obtained with eight other individuals with intellectual disabilities (McIlvane et al., 1992, Experiment 1), using a similar procedure. The subjects named an undefined visual stimulus after a number of exposures to exclusion trials, ranging from three to 15.

The emergence of naming after exclusion trials was also verified by Costa, Grisante, Domeniconi, de Rose, and de Souza (2013), in a study conducted with eight preschool children with typical development. The aim of this study was to determine the required number of exclusion trials to learn two undefined name-figure relations. The tasks, conducted on a touch screen computer, involved the presentation of the undefined stimuli through exclusion trials, followed by learning and naming probes. If the naming was not verified, the exclusion trials were repeated and the tests carried out again until the correct naming of the undefined figures was achieved. In this study, both the exclusion trials, as well as the naming and learning probes, were performed in extinction. All children correctly completed all the exclusion trials, requiring between three and 10 trials to name the new stimuli, a number similar to that found in the study of McIlvane and Stoddard (1981) and McIlvane et al. (1992) with individuals with intellectual disabilities.

Therefore, it can be assumed that new auditory-visual conditional relations can be consistently taught through exclusion trials and the emergence of naming of the new visual stimuli can be observed from this procedure. However, the required number of exposures to exclusion trials proved to be variable in the studies presented, both in the analysis of a single subject that learned several relationships and in the analysis of groups of individuals (with intellectual disabilities or typical development). It is possible to speculate that the learning of new name-object relations can vary depending on at least two variables: the teaching procedure used and the repertoire of prerequisites of the participant. The first variable was directly investigated by Langsdorff, Domeniconi, and Schmidt (2015).

Aiming to achieve a stable number of exclusion trials at teaching auditory-visual relations, Langsdorff et al. (2015) developed a procedure involving manipulations highlighted in the literature as favorable to learning via exclusion trials: use of manipulable stimuli (Domeniconi et al., 2007); use of reinforcement contingent on responding by exclusion (Carr, 2003; Ferrari et al., 1993, 2008; McIlvane & Stoddard, 1981); more than one exposure to the exclusion trials prior to learning tests—two exclusion trials were presented before the tests (McIlvane et al., 1984; Wilkinson et al., 2009); and teaching of one relation at a time, with tests to guarantee the learning before the introduction of the next relation to be taught (McIlvane et al., 1992). Participants were eight children with typical development, aged 5 to 9 years; all the children learned the four auditory-visual relationships taught, six of them did so with the minimum number of trials programmed by the procedure (two) and two other children needed four exposures for only one of the relations taught. In the naming probes, five children correctly named the four undefined visual stimuli after the two exposures, two children named three stimuli, and one child correctly named only one of the stimuli after two exposures. These results were more positive than those of the study of Costa et al. (2013) and suggest that the number of exclusion trials required for learning may differ depending on the procedure used, as the manipulations used in the study by Langsdorff et al. (2015) were able to produce learning after fewer exclusion trials. It is still necessary to clarify whether these data can be replicated with people with developmental delays and different educational repertoires.

The present study aimed to investigate whether, from the application of the teaching procedure used by Langsdorff et al. (2015) with participants with autism and Down syndrome and different educational histories, it would be possible to establish a stable number of exclusion trials necessary for teaching auditory-visual relations to individuals with developmental problems. In addition, this study aims to verify the occurrence of the emergency of naming after the teaching of auditory-visual relations by exclusion, as obtained by McIlvane et al. (1984).

## Methods

### Participants

Participants were 14 individuals (10 males) aged 5 to 46 years: three adults with Down syndrome and 11 individuals (children and adults) with a previous diagnosis of autism. The participants with autism were divided into two groups. The first group (EI) consisted of seven subjects, aged 5 to 17 years. All had a history of early behavioral intervention, based on the ABA principles, with individualized curriculums constructed from behavioral evaluation data (CARS, PEP-R). All these participants, except EI7, attended regular schools. All used speech to communicate, although with varying levels of fluency. The second group of individuals with autism (NI) was comprised of four young people and adults, aged 16 to 39 years, who attended a special education institution but had no history of individualized intervention. In the institution, they attended group activities for the development of daily living skills (e.g., cooking, manual tasks, self-care) and to learn a basic repertoire of reading through a computer program, which included matching to sample tasks. Two of these participants did not speak. All the participants with Down syndrome (28 to 46 years of age) used speech to communicate and, due to being adults, attended the same special education institution as the NI group.

The scores of the children with autism and early intervention in the ABLA (Assessment of Basic Learning Abilities) test (Kerr, Meyerson, & Flora, 1977), CARS (Childhood Autism Rating Scale) (Schopler, Reichler, DeVellis, & Daly, 1980), and PEP-R (Psychoeducational Profile Revised) (Schopler, Reichler, Bashford, Lansing, & Marcus, 1990) are presented in Table 1. Table 1 also presents the level of support needed by the adults who participated on this study according to the Support Intensity Scale (SIS), described by the American Association on Intellectual and Developmental Disabilities (AAID) as a resource to categorize the intensity of support required by individuals with intellectual and developmental disabilities along their everyday living (Smith & Tyler, 2010).

**Table 1:** General characteristics of the participants

Participant	Gender	Age (years)	Speech	CARS score	PEP-R score	SIS
EI1	M	5	Yes	31.5 (mild/moderate)	82 (2 years 10 months)	–
EI2	M	6	Yes	27.5 (normal)	105 (4 years)	–
EI3	M	7	Yes	36.5 (mild/moderate)	101 (3 years 10 months)	–
EI4	M	8	Yes	30 (mild/moderate)	109 (4 years 3 months)	–
EI5	M	10	Yes	26 (normal)	109 (4 years 3 months)	–
EI6	M	11	Yes	33 (mild/moderate)	105 (4 years)	–
EI7	F	17	Yes	–	–	Extensive
NI1	M	16	No	–	–	Pervasive
NI2	M	24	Yes	–	–	Extensive
NI3	F	26	Yes	–	–	Pervasive
NI4	M	39	No	–	–	Pervasive
DS1	F	28	Yes	–	–	Intermittent
DS2	F	38	Yes	–	–	Intermittent
DS3	M	46	Yes			Intermittent

Participants with autism with an early behavioral intervention history are identified by the abbreviation EI, participants with autism without an early behavioral intervention history are identified by the abbreviation NI, and participants with Down syndrome are identified by the abbreviation DS

The parents were informed about the research aims and the tasks to be performed and, prior to the start of the data collection, signed a consent form, authorizing the participation of their children, conforming to Brazilian law.

### Materials and stimuli

The different phases of the procedure (four) were organized in notebooks with different colors in the following order: white, yellow, blue, and green. The different colors were used to facilitate the management and organization of the material by the researcher. The trials, organized sequentially in the notebooks, were constructed on 105 mm × 145 mm pages (half an A4 sheet). At the top of each page was an empty square with a piece of Velcro filling its interior. There were also three manipulable figures fixed to a Velcro strip at the page’s bottom; each of these figures could be removed and fixed to the Velcro of the empty square at the page’s top.

Defined and undefined stimuli were used throughout the different phases. The defined stimuli were the figures and the respective dictated words cat, house, and ball. The undefined stimuli consisted of the dictated words Pagu, Mido, Fani, and Duca and their respective figures, as well as also undefined figures and words used in the test phases, as shown in Table 2.

**Table 2:**
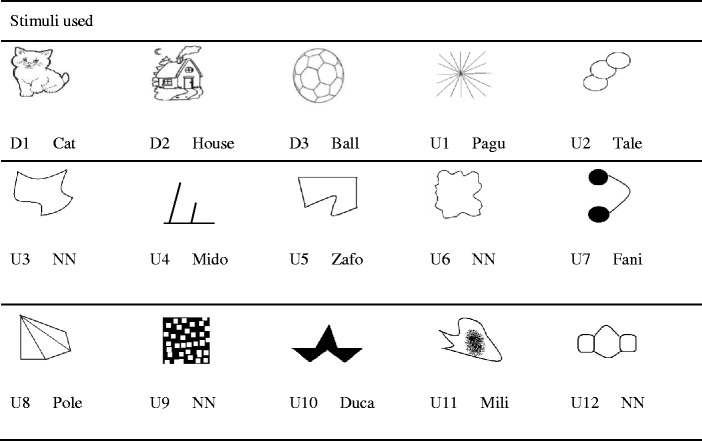
Auditory and visual stimuli used in the study

The figures are accompanied by the corresponding names, preceded by the letter D (defined) or U (undefined). NN = stimuli without assigned names. U1, U4, U7, and U10 were presented through exclusion trials, and the other undefined stimuli were only presented in the learning tests

### Procedures

This study was approved by the Human Research Ethics Committee of the Federal University of São Carlos (CAAE authorization No. 0357.0.135.000.11). Individual sessions were carried out in a classroom of the school attended by the participant. The environment consisted of a table, two chairs, and a camcorder. The participant sat next to the experimenter during the activities. All the participants performed the complete procedure in a single session, with an average duration of 20 min. Matching to sample trials were performed in which the sample stimulus was auditory (dictated word) and the comparison stimuli were visual (figures). Thus, presented with questions such as “What is the cat?” or “Where is the cat?”, the participant should take one of the three figures at the bottom of the notebook page and fix it to the top, inside the empty square.

Correct responses were followed by praise in all the phases, except in the learning tests. For some children (EI1 and EI2), cards of a cartoon character were also used as boosters at the end of each block, not contingent on performance, only on participation. Incorrect responses were followed by a correction procedure in the training steps, involving the emission of the verbal response “no” by the experimenter and the repositioning of the stimuli for the repetition of the trial.

The stimuli presented in the exclusion trials were always the dictated names Mido, Pagu, Duca, and Fani and their corresponding figures; however, the order in which they were presented to the participants varied. The number of exposures, order, and position of the stimuli presented in all the study trials followed the criteria presented by Green (2001) to guarantee the establishment of conditional relations. The experiment was conducted in six phases, as described below.

#### Phase 1—baseline of auditory-visual relations

In this phase, the auditory-visual matching to sample task was taught/strengthened for the participants using three known relations (ball, house, and cat). A block of nine trials was conducted, and each familiar stimulus was dictated three times. The criterion for passing onto the next phase was 100% correct responses in the block. In case of an error in at least one trial, the block was repeated.

#### Phase 2—introduction of the blank comparison as the neutral stimulus

In this phase, participants were taught to search for stimuli hidden by a “mask” (blank comparison), an important repertoire for the performance in the posterior learning tests. The use of the blank comparison in this study did not have the function of determining stimulus control routes; this technique was used to replicate the tests of the study by Wilkinson and McIlvane (1997), which was also used by Costa et al. (2001) and Domeniconi et al. (2007), aiming to allow an increased number of possible responses by not reproducing forced choice trials. The introduction of the blank comparison as the neutral stimulus took place over one block of 12 trials, in which one of the three comparison stimuli was gradually covered until it became completely opaque. In the first two trials, the correct stimulus was covered with transparent paper, allowing a clear view of the figure behind it. In the two following trials, the correct stimulus was covered by two layers of transparent paper. This was followed by the correct stimulus being covered with semi-opaque paper for two trials. Finally, a cover consisting of two layers of semi-opaque paper completely hid the stimulus in the fifth and sixth trials. In the next four trials, the position of the blank comparison was alternated between the correct stimulus and an incorrect stimulus. The criterion for passing to the next phase was 100% correct responses in the block.

#### Phase 3—exclusion trials with a pair of undefined stimuli

The aim of this phase was to expose the participants to new pairs of auditory-visual stimuli through teaching by exclusion trials. A pair of undefined stimuli (a dictated word and a figure) was presented through trials involving the presentation of familiar stimuli as the samples and comparisons. The block was composed of eight trials, with six baseline and two (fourth and eighth trials) with a pair of undefined stimuli. The blank comparison was present in all trials, except those in which the undefined stimuli were presented. In the exclusion trials, a new word was dictated (e.g., Mido), and the comparison stimuli were two familiar figures and one undefined one. Praise was contingent on correct responses, as in the earlier phases, including the exclusion trials. The criterion to advance to the next phase was the selection of the undefined figure when presented with the new name dictated in the two trials.

#### Phase 4—learning tests of the relations between name and figure

In this phase, the aim was to verify whether the procedure had been able to teach the participants the relation between the undefined dictated name and the undefined figure presented in the exclusion trials. When learning was not observed, the aim was to quantify the number of exclusion trial repetitions that were necessary for learning to occur. Four learning probes were conducted, as presented in Table 3, using the example of the auditory stimulus Mido and its respective figure.

**Table 3:**
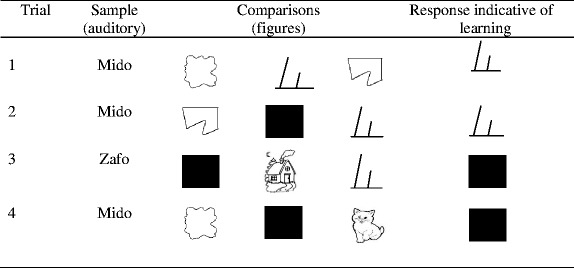
Examples of the learning tests from phase 4

In the first learning test, the undefined name presented in the previous phase (e.g., Mido) was the sample and, as comparisons, the figure related to the word, available in the exclusion trials, and two other undefined figures. This first test, which aimed to evaluate the possible novelty-control of the response, was not part of the test suite used by Wilkinson and McIlvane (1997) and was added to this study in order to allow the presentation of two undefined stimuli simultaneously to the stimulus presented in the exclusion trials. The response was considered correct when the stimulus presented in the exclusion trials was selected.

The second learning test used the undefined word presented in the previous phase as the sample stimulus (e.g., Mido), and the figure corresponding to the word, an undefined figure and the blank comparison as the comparisons. The aim of this test was to verify whether the participant selected the figure presented in the exclusion trials, even having the possibility of rejecting the available visible comparisons, selecting the blank comparison—which would be analogous to saying that none of the figures corresponded to the dictated name; furthermore, the participant could also select the new undefined figure, suggesting novelty-control.

The third learning test consisted of presenting a completely new word (e.g., Zafo) as sample, and the blank comparison, a familiar stimulus and the figure presented in the exclusion trials as the comparisons (e.g., Mido). The response indicative of learning was the selection of the blank comparison, which indicates that the participant identified the figure presented in the exclusion trial as a defined stimulus and therefore not corresponding to the new undefined name dictated in the trial. Conversely, the selection of the figure presented in the exclusion trials when presented with the undefined stimulus sample indicates that, for the participant, the figure maintained the status of undefined stimulus and that the relation between the dictated word and figure had not been established through exclusion trials.

The fourth learning test used the word presented in phase 3 as the sample (e.g., Mido) and the blank comparison, a familiar figure and an undefined figure as the comparison stimuli. The aim of this test was to verify whether, in the absence of the figure presented in the exclusion trials—hidden under the blank comparison—and having an undefined figure available, the participant would select the blank comparison when presented with the name dictated in the exclusion trials.

When a participant responded incorrectly to any of the tests, phase 3 was performed again, followed by the learning tests (phase 4) being reapplied. The phase was finished when either the participant responded correctly in all learning tests or after 10 repetitions of phases 3 and 4, whichever occurred first. Therefore, considering that the procedure could potentially take a long time—given the criteria of 10 repetitions for the closure of this phase—baseline trials between the tests were not introduced. Reinforcers contingent on correct responses or participation in the activity were not used in this phase. Thus, after fixing the figure chosen by the child to the page of the trial, the page would be turned and the following trial started, without providing differential consequences programmed for the responses considered correct or incorrect.

#### Phase 5*—*naming test

The aim of this phase was to assess whether, once learning of the new auditory-visual relation in phase 4 had been demonstrated, the participant would name the new stimulus. If the participant did not demonstrate learning in phase 4, they would return to phase 3 without being exposed to the naming test. The performance in the naming test was not part of the criteria to complete this phase and start the training with a new pair of stimuli. The individual performance was recorded on video for later analysis; however, regardless of correct or incorrect performance, the participant received praise (for cooperation in the activity) and progressed in the procedure.

#### Phase 6*—*replication

The procedures described in phases 3, 4, and 5 (exclusion trials, learning tests, and naming tests) were replicated with three other pairs of stimuli (three undefined names were related to three other undefined figures). The order of presentation of each pair for each participant was random.

### Data analysis procedure

Data were analyzed so as to quantify the number of trials required for the participants to emit responses indicative of learning in the four test trials, performed with each of the new stimulus pairs. The performances in the naming tests recorded on video were also analyzed. The reliability of the results of the naming tests, calculated by dividing the number of agreements between two observers by the sum of agreements and disagreements, multiplied by 100, was 100%.

## Results

### Baseline

In phase 1, all participants, with the exception of NI1, demonstrated 100% accuracy in the baseline trials of auditory-visual conditional discriminations and advanced to the next phase after only one block of trials. Participant NI1 took three blocks to advance to the next phase—55% correct responses in the first block, 33% correct responses in the second block, and 100% correct responses in the third block.

### Introduction of the blank comparison as the neutral stimulus

All participants learned to select the blank comparison as the neutral stimulus when none of the comparison stimuli corresponded to the sample stimulus. All participants, except NI1 and NI2, achieved the criterion of 100% correct responses with a single exposure to this block. Participant NI1 achieved the criterion after three exposures, obtaining 91% correct responses in the first block, 75% correct responses in the second block, and 100% correct responses in the third block. Participant NI2 completed the phase in two blocks, with 83% correct responses in the first and 100% correct responses in the second.

### Exclusion trials and learning tests

All participants with autism and EI presented performance by exclusion in all the trials presented and learned the four auditory-visual relations. Variability was observed in the amount of exposures to the exclusion trials for the learning criterion to be achieved (minimum of two and maximum of 20 exposures). Four of the seven participants with EI demonstrated learning of the four pairs of stimuli with only two exposures (the minimum programmed in the procedure); two participants learned the relations of two pairs of stimuli with the minimum number of programmed trials, and only one required 10 exposures for learning one of the two pairs, however, achieved the learning criterion for the other pair with two or four exposures. Despite the variation found, it was observed that the data were predominantly concentrated in the minimum number of repetitions (two—see Table 4).

**Table 4:** Number of exclusion trials needed for correct responses in all learning tests (LT) and results of the naming tests (N)

Participant	Mido		Pagu		Fani		Duca	
	LT	N	LT	N	LT	N	LT	N
EI1	6	√	2	“titi”	4	√	2	“guga”
EI2	4	√	2	√	10	√	4	√
EI3	2	“don’t know”	2	√	2	√	2	√
EI4	2	√	2	√	2	√	2	√
EI5	2	√	2	√	2	√	2	√
EI6	2	√	2	√	2	√	2	√
EI7	4	√	2	√	8	√	2	√
NI1	Did not respond by exclusion							
NI2	Did not demonstrate learning							
NI3	2	√	2	√	2	√	2	√
NI4	Did not demonstrate learning							
DS1	6	√	2	√	8	√	2	√
DS2	2	√	2	√	2	√	2	√
DS3	2	√	2	√	2	√	2	√

*EI* participants with autism and early intervention, *NI* participants with autism and no early intervention, *DS* participants with Down syndrome

All four participants of the NI group responded by exclusion, except NI1. This participant, even after the correction procedure, selected one of the defined comparison stimuli when given an undefined dictated name; due to this performance, NI1 did not perform the learning and naming tests. Only one participant (NI3) demonstrated learning of the auditory-visual relations after exclusion trials, with the minimum number of planned exposures. Participants NI2 and NI4, despite having responded by exclusion, did not show learning of any of the stimuli pairs after the maximum number of repetitions of phase 3 (20 exposures) and therefore did not go on to do the naming tests.

All the participants with Down syndrome responded by exclusion and learned the four auditory-visual relations. Two of them (DS2 and DS3) learned the four pairs of relations after two exclusion trials. Participant DS1 learned two pairs (Pagu and Duca) after two exclusion trials and the other two pairs with six and eight exposures (Mido and Fani, respectively).

### Naming tests

Of the seven participants with autism and EI, five correctly named the taught visual stimuli after achieving the criterion in the learning probes (Table 4). Participant EI3 did not name only one of the stimuli (Mido) and EI1 named two stimuli incorrectly. For these participants, the procedure was effective in producing naming performance, which was not directly taught. Very similar data were obtained with the participants with Down syndrome: all three were able to name all the visual stimuli, after achieving the proposed learning criterion. The only participant with autism without EI that demonstrated positive results in the learning probes (NI3) correctly named all the visual stimuli.

## Discussion

This study aimed to investigate whether, from the application of the teaching procedure used by Langsdorff et al. (2015) with participants with autism and Down syndrome and different educational histories, it would be possible to establish a mean number of exclusion trials necessary for learning these relations. In addition, this study sought to verify whether, from the teaching by exclusion of auditory-visual relations, the naming repertoire of the stimuli taught would emerge, as obtained by McIlvane et al. (1984).

To achieve these aims, different to the procedures commonly used to test the learning of auditory-visual relations after exclusion trials (e.g., Dixon, 1977; Carr, 2003; Wilkinson & McIlvane, 1997), four different test trials were presented for each pair of stimuli taught. Each trial checked different possibilities of control for the responses in order to ensure that the name-figure relation established in the exclusion trials had been learned. The participant was presented to the naming test only after successfully completing these four trials.

All participants, except one, presented performance by exclusion; additionally, all participants with autism of the EI group, as well as the participants with DS, presented learning of all the pairs of stimuli taught. The majority of these subjects (DS and autism with EI–six out of 10) learned all the pairs of stimuli with a minimum number of programmed exclusion trials; three other participants learned at least two pairs of stimuli with the minimum number of exposures. These results indicate that the procedure was efficient to promote learning in these participants, from a reduced number of trials. In addition, these data are comparable to those obtained with participants with normal development in the study of Langsdorff et al. (2015).

The learning performances observed with these participants, in general, replicate other studies in which exclusion trials were successfully used as a teaching procedure (e.g., Ferrari et al., 1993, 2008; McIlvane & Stoddard, 1981; McIlvane et al., 1984; McIlvane et al., 1992; Wilkinson et al., 2009). Furthermore, the difficulty of learning arbitrary relations for people diagnosed with autism spectrum disorders, widely indicated in the literature (Dube & McIlvane, 1995; Eikeseth & Smith, 1992; Gomes & de Souza, 2008; Gomes, Varella, & de Souza, 2010; Kelly, Green, & Sidman, 1998; Vause, Martin, Yu, Marion, & Sakko, 2005; Williams, Pérez-González, & Queiroz, 2005), was not confirmed by this study, considering the data from the EI group. These positive results can be attributed to the set of conditions prepared in the procedure, all recommended by studies on teaching by exclusion trials, and the presence of important repertoires for the performance of the proposed tasks in the participants. The individuals with autism of the EI group and the participants with Down syndrome obtained a score 6 in the ABLA test, which indicates that they were able to correctly perform combined auditory-visual discrimination tasks (Verbeke, Martin, Yu, & Martin, 2007). The performance of individuals in the ABLA test is a reliable predictor of the type of task they are able to perform (Martin, Yu, & Vause, 2004), which means that the participants of this study had sufficient repertoires to perform the tasks proposals in the procedure (select a figure faced with the presentation of an auditory sample). Additionally, it must be considered that participants with autism of the EI group attended regular schools at the time of conducting the study, or had done so previously. Exposure to typical activities performed at school may involve similar academic situations to those conducted in the study, favoring the performance of the proposed tasks.

Conversely, the participants with autism of the NI group presented notably lower performance than the other participants. Only one of them (NI3) showed learning of the relations, although it should be noted that this participant learned the four relations after only two exclusion trials. The other participants either did not present performance by exclusion (NI1) or did not demonstrate learning of the pairs of stimuli, even with 20 exposures to exclusion trials for each pair (NI2 and NI4). The participants of this group (NI) were all adults and had no history of early behavioral intervention. Participants NI1 and NI4 obtained a score of 4 in the ABLA test, which means that their ability to discriminate visual-visual relations would be present in their repertoire; however, the ability to discriminate auditory-visual relations would be absent. This absence of the repertoire required for the task of this experiment can be directly related to the difficulty of NI1 to achieve the learning criterion defined for the relations (cat, dog, and house) and to demonstrate performance by exclusion in the teaching trials. Similarly, this lack of established repertoire for the performance of auditory-visual conditional discriminations may have negatively interfered in the learning of the relations taught for NI4.

The results of NI1, NI2, and NI4 (NI group) suggest, as indicated by McIlvane and Stoddard (1981) and McIlvane et al. (1984), the importance of the presence of prerequisite skills for the type of procedure proposed. With the data presented here, it is not possible to say which skills were absent in these individuals, making it impossible for them to successfully complete the task. In addition to the difficulties in the auditory-visual discrimination tasks, previously described, it is possible that, in the case of NI2 and NI4, the correct performance in the exclusion trials was merely established by the incorrect comparison, which would not support successful performance in the learning tests. However, this assumption cannot be supported by the data presented here, although it indicates the need to clearly establish what skills must be present in the repertoire of subjects before implementing the type of procedure proposed here. It should also be mentioned that despite the age differences between participants of the NI group and EI group, this does not seem to be a critical variable considering the performances of the DS group. Both participants of NI group and of DS group were adults; nevertheless, the performances of participants of DS group were similar to the performances of participants of the EI group. This data suggests that the early intervention, not the age, seems to be the critical variable (Baer, 1970).

Regarding the naming performance, it was found that all the participants who demonstrated learning of the relations taught in the learning probes were able to name the undefined visual stimuli. Only two participants, EI1 and EI3, were unable to correctly name all the trained stimuli; however, they were able to name at least two of them (the teaching order of the pairs was not the critical variable in the difficulty of these participants: Participant EI1 did not name the third and fourth stimuli taught, and EI3 did not name the first visual stimulus taught). These data extend the findings of McIlvane et al. (1984, 1992), obtained with participants with intellectual disabilities, indicating that the teaching of a repertoire of auditory-visual discrimination could encourage the emergence of the repertoire of naming, even in individuals with different developmental problems. Although the listener and speaker repertoires are independent, this procedure seems to have favored the selection of undefined figures when presented with the spoken name (listener repertoire) in the learning tests, as well as the naming of the undefined stimulus (speaker repertoire) in the naming tests, as in other studies with different populations (e.g., de Souza et al., 2009).

Nevertheless, it must be considered that the required number of exposures to exclusion trials for the emergence of naming should be analyzed with caution. Despite the majority of the study participants correctly naming the stimuli after two exposures to exclusion trials (a more stable number and generally lower than that obtained in the studies by McIlvane et al., 1984, 1992), it should be highlighted that, in addition to hearing the name of the stimuli in these two opportunities, these names were repeated three more times in the learning tests. Thus, the undefined names were associated with the new figures at least five times prior to the application of naming tests. This number of exposures is close to the mean number of exposures necessary for the correct naming of the new stimuli (six) registered by Costa et al. (2013), with it not being possible to evaluate whether only two exposures through exclusion trials would be sufficient for the correct naming of the stimuli.

The results of this study are relevant for future investigations into the possibility of maintaining the learned response in the context of teaching by exclusion over time. The issue of maintenance of the repertoire learned through this procedure is especially important when considering the possibility of its wider use in applied contexts. The teaching of new and functional words is important for individuals with autism or other language difficulties. The construction of teaching procedures that promote rapid learning at low cost—as is the case of this study—not only of nouns but also of adjectives and verbs can be considered a priority. Future studies to determine the feasibility of this procedure in applied contexts can make use of the material described here—including the tests of repertoire maintenance and generalization for new environments—and can be conducted in institutions specialized in teaching individuals with difficulties in language acquisition.

## Conclusion

Despite the fact that many issues of this study need further investigation, it is important to highlight that exclusion teaching procedure has proven itself to be an effective tool to teach relationships between stimuli in a fast and robust manner across a wide learning profile range of individuals.
